# Notes and News

**Published:** 1904-06-25

**Authors:** 


### Notes and News.

The plague has appeared again in several districts in Egypt.

The Metropolitan Hospital Sunday Fund Collection now exceeds £19,000.

The Bath Club will give an entertainment in aid of King Edward's Hospital Fund on July 4th.

The late Miss Mary Young, of South Shields, has bequeathed £11,000 to the South Shields Infirmary.

St. Bartholomew's Hospital has benefited to the extent of £609 through the concert recently given by Signora Giulia Ravogli.

The Prizes to the Students of the Royal Dental Hospital of London will be presented by Sir William Collins, F.R.C.S., on Wednesday, July 13, at 8 p.m.

The Society for the Prevention of Cruelty to Children will benefit to the extent of nearly £2,000 from the Children's Fête, recently held in the Albert Hall.

The late Mrs. Elizabeth Withams, of Putney, has bequeathed £500 to the Cancer Hospital, and £200 each to the London and the Poplar Hospitals, and £100 to the Victoria Hospital, Romford.

The late Mr. Charles Palmer, of Lee, has bequeathed a small legacy to each of the following institutions:—The London, the Cancer, the City of London for Diseases of the Chest, and the Gravesend Hospitals, also to the Bromley Cottage Hospital and the East London Hospital for Children.

The new Isolation Hospital has been opened at Rothwell for the Rural and Urban District Councils of Hunslet, Rothwell, and Methley. The buildings to accommodate 28 patients are erected on a site of five acres. The architect is Mr. W. E. Richardson, of Rothwell, and the cost is estimated at about £10,000.

A Home for the Dying has been opened in Dublin, through the benevolence of an anonymous donor, who gave £5,000 towards the scheme, which will benefit 24 patients.

The Chalmers Hospital, Edinburgh, will be closed from June 30th to October 1st, to permit of the extensive alterations and improvements which are about to be undertaken. These include a new out-patients' department and operating theatre.

At the last meeting of the Senate of the University of Dublin degrees were conferred for the first time on women, and now women are on an equality with men as far as degrees in Arts and Medicine are concerned, but they cannot become Fellows or Scholars on the Foundation.

Germany has carried off the Warren Prize of £100, in the gift of the Massachusetts General Hospital, for the best dissertation on a physiological, surgical, or pathological subject. The prize winner was Dr. Max Borst, Professor in Pathological Anatomy at the University of Wurzburg.

With the increase in knowledge, new dangers surrounding our milk supply are constantly brought to light. Within a brief period our attention is drawn to epidemics of sore throat traceable to milk, and to a statement that 15,000 gallons of formalin have been sold to milk vendors in one year.

The Queen attended the Bazaar in Aid of the Victoria Hospital for Children held in the Royal Albert Hall yesterday. She remained some time, making numerous purchases. On her return to Buckingham Palace she caused a letter to be sent expressing her appreciation of the arrangements and wishing the hospital success.

A monthly collection scheme of one penny, which has proved very successful in Herefordshire, is about to be adopted in Worcestershire on behalf of local hospitals. The scheme provides that in each parish or group of parishes a committee is formed, which appoints honorary collectors, the number being in proportion to the population.

The Italian Umberto first prize for the best work or invention relative to orthopædic surgery is open to medical men of all nationalities. The prize, amounting to £140, is awarded by the Rizzoli Orthopædic Institute of Bologna, and competitors should communicate with the President at the institute. The competition closes on December 31st.

The value of prompt measures and precautions in dealing with an outbreak of small-pox is demonstrated by a recent experience at Rowton House. Of a daily average of 350 residents, only 26 caught the disease. Re-vaccination was offered to every inmate, accompanied by a gratuity of two shillings, but very few accepted the offer. No new unvaccinated lodger was admitted, and of the 40 officials, all of whom had been re-vaccinated, not one contracted small-pox.

